# Older adults’ participation and experiences in design thinking for AI-driven assistive technology: a scoping review

**DOI:** 10.1186/s12877-026-08096-0

**Published:** 2026-08-10

**Authors:** Dhika Dharmansyah, Laili Rahayuwati, Iqbal Pramukti, Kuswandewi Mutyara

**Affiliations:** 1 Doctoral Study Program in Medical Science, Faculty of Medicine, Universitas Padjadjaran, Sumedang, West Java Indonesia; 2 Department of Nursing, Faculty of Sport and Health Education, Universitas Pendidikan Indonesia, Bandung, West Java Indonesia; 3 Department of Community Health Nursing, Faculty of Nursing, Universitas Padjadjaran, Sumedang, West Java Indonesia; 4 Department of Public Health, Faculty of Medicine, Universitas Padjadjaran, Sumedang, West Java Indonesia

**Keywords:** Older adults, Design thinking, Assistive technology, Artificial intelligence, Co-design; participatory research, Healthy ageing, Digital health, Ageing in place, Scoping review

## Abstract

**Background:**

Population ageing and the rapid integration of artificial intelligence (AI) into assistive technologies have created a need to engage older adults as active partners in technology design. AI-driven assistive technologies are frequently developed with limited older-adult involvement, a pattern associated with low adoption, poor fit with daily routines, and the reinforcement of ageist assumptions about older adults as passive recipients of care. Design thinking (DT)—an empathy-driven, iterative, human-centred methodology—offers a structured framework for engaging older adults as co-designers. To date, no scoping review has mapped how older adults participate in DT processes for AI-driven assistive technology, or the ethical and experiential dimensions of that participation.

**Methods:**

This scoping review followed Arksey and O’Malley’s five-stage framework with Levac et al. refinements and was reported in accordance with the PRISMA extension for Scoping Reviews (PRISMA-ScR). The protocol was prospectively registered on the Open Science Framework (https://doi.org/10.17605/OSF.IO/8W2S5). Systematic searches across Web of Science, IEEE Xplore, Scopus, and PubMed/MEDLINE (10–16 March 2026; updated 20 June 2026) identified 176 records. Following deduplication (n = 146) and two-stage independent screening, 15 studies were included. Data were charted in duplicate using a piloted extraction form extended with experiential and ethical dimensions, appraised descriptively (JBI; MMAT), and synthesised through inductive thematic synthesis.

**Results:**

The 15 included studies (2015–2025) spanned seven countries and a range of AI-driven assistive technologies, including large language model (LLM)-based conversational agents and companion robots, smart-home sensing and fall-detection systems, embodied conversational agents, and AI-enabled wearables. Thematic synthesis generated four analytical themes: (1) uneven participation across the DT cycle, concentrated in the empathise and test phases; (2) facilitators and barriers to meaningful participation; (3) a continuum of agency from tokenistic consultation to genuine co-design; and (4) structural and ethical conditions shaping inclusion. Evidence gaps were most pronounced for older adults with cognitive impairment and for older adults in low- and middle-income countries (LMICs).

**Conclusions:**

Meaningful participation of older adults in DT for AI-driven assistive technology is methodologically and ethically important for producing acceptable, usable, and contextually appropriate technology. This evidence map offers guidance for geriatric researchers, clinicians, digital-health implementers, and policymakers committed to inclusive co-design with older adults, and identifies priority gaps for future primary research.

**Trial registration:**

Open Science Framework https://doi.org/10.17605/OSF.IO/8W2S5

**Supplementary Information:**

The online version contains supplementary material available at https://doi.org/10.1186/s12877-026-08096-0.

## Background

The world’s population is ageing at an unprecedented pace. By 2030, one in six people worldwide will be aged 60 years or over, a proportion projected to correspond to 2.1 billion people by 2050 [1]. This demographic transition has created urgent imperatives for health and social-care systems to support older adults’ independence, safety, functioning, and quality of life [1, 2]. Artificial intelligence (AI)-driven assistive technologies—encompassing conversational agents, fall-detection systems, cognitive-assistance tools, smart-home automation, and AI-enhanced mobility aids—have increasingly been identified as promising responses to these imperatives [3–5]. It is important to note that assistive technology is a broad category, much of which contains no AI component; the present review concerns specifically the subset in which an AI capability (for example, machine-learning-based activity recognition, natural-language understanding, or adaptive personalisation) is integral to the device’s assistive function. For example, an “AI-enhanced mobility aid” denotes a walker or wheelchair augmented with machine-learning-based fall-risk prediction or adaptive navigation, as distinct from a conventional, non-AI mobility aid.

Despite their promise, AI-driven assistive technologies frequently fail to achieve adoption or sustained use by older adults. Across the broader assistive-technology and gerontechnology literature, a substantial minority of devices are abandoned within the first year of use, with reported non-use and abandonment commonly in the region of 30% [6, 7]. A recurring explanation concerns the design process itself: older adults—the people whose lives these technologies are intended to support—are frequently engaged late, narrowly, or not at all as active partners in development [3, 8]. When end-users are not meaningfully involved, the resulting technologies risk misaligning with users’ actual capabilities, preferences, routines, and health priorities [7]. These misalignments carry tangible consequences for older adults: unmet care needs, reduced autonomy, abandonment of potentially beneficial devices, and the reinforcement of ageist assumptions that position older adults as passive recipients of technology rather than as active partners in shaping the tools intended for their use [9].

Ageism—systematic stereotyping of, prejudice toward, and discrimination against people on the basis of age [10]—operates as a structural barrier to inclusive participation in technology design [8, 9]. Its effects are particularly consequential in AI development, where biased training data and ageist design assumptions can be encoded into deployed systems at scale [9]. International policy increasingly recognises that meaningful end-user involvement in technologies that affect older adults is not optional. The United Nations (UN) Convention on the Rights of Persons with Disabilities affirms the right of persons—including older adults—to participate in decisions affecting them [11]. The United Nations Educational, Scientific and Cultural Organization (UNESCO) Recommendation on the Ethics of Artificial Intelligence calls for inclusive participation of stakeholders throughout the AI lifecycle [12]. The European Union (EU) Artificial Intelligence Act introduces a risk-based framework with enhanced human-oversight and transparency obligations for higher-risk systems, including many that serve older or vulnerable users [13]. The Institute of Electrical and Electronics Engineers (IEEE) Ethically Aligned Design framework stipulates that AI design processes should respect the autonomy, dignity, and agency of intended users [14].

Participatory and user-centred approaches are increasingly recognised as both methodologically effective and ethically necessary for producing assistive technologies that are acceptable, usable, and contextually relevant to older adults [7, 8]. Among these approaches, design thinking (DT) occupies a distinctive position. DT is an iterative, empathy-centred problem-solving methodology encompassing five non-linear phases: empathise (deep immersion in users’ experiences), define (problem framing), ideate (creative generation of options), prototype (rapid materialisation of concepts), and test (iterative evaluation with users) [15, 16]. What distinguishes DT from adjacent human-centred and participatory frameworks is its explicit positioning of empathic immersion as both the starting point and the organising principle of the entire process: the empathise phase is not a preliminary requirements-gathering step but a sustained orientation that recurs across the cycle, privileging users’ lived experience as the primary source of design insight [17, 18]. Applied with older adults in AI-driven assistive technology development, DT therefore constitutes both a methodological choice and a commitment to treating older adults as experts in their own lived experience and as active partners in design.

The application of human-centred design in health research has expanded rapidly. In a scoping review of 21 studies, Bazzano and colleagues [19] found that human-centred design in global health was applied most often in early, formative phases (needs assessment and concept generation), that end-user engagement was frequently concentrated in initial empathy and ideation activities rather than sustained across implementation, and that reporting of methods and of the depth of participation was inconsistent—observations that foreshadow the participation patterns examined in the present review. Göttgens and Oertelt-Prigione [20] reviewed 82 studies and confirmed the widespread but heterogeneous use of human-centred design approaches in digital health, again noting variable depth and reporting of participation. Rivera and colleagues [18] published a scoping review protocol examining DT in physical-activity research. Cho and colleagues [3] examined older adults’ engagement in the design of AI health technologies broadly; however, they did not adopt a DT lens, did not examine older adults’ subjective experiences within DT-specific processes, and did not analyse the ethical and experiential quality of that engagement.

These prior reviews establish that older adults are sometimes included in technology design. However, they leave unexamined how that inclusion is experienced and whether it is ethically substantive. These are precisely the questions that matter most for geriatric practice. The ethical and experiential lens of the present review is therefore not an abstract concern. For older adults, the quality of participation directly shapes whether a technology respects their autonomy and dignity, fits their everyday routines, and is ultimately adopted rather than abandoned. Superficial or tokenistic involvement tends to produce tools that are declined or discarded, with downstream consequences for safety, independence, and equitable access to care. These stakes are amplified in developing-country and resource-constrained settings, including Indonesia. In these settings, population ageing is accelerating, care ecologies differ markedly from the high-income contexts in which most devices are designed, and importing technologies developed elsewhere without local co-design risks poor fit and inequitable benefit. Documenting the experiential and ethical landscape is therefore a prerequisite for designing AI assistive technologies that are not only usable but just.

To date, no scoping review has mapped how older adults participate in DT processes for AI-driven assistive technology development, together with the ethical and experiential dimensions of that participation. Without a systematic evidence base documenting how participatory practices operate—or fail to operate—in DT processes with older adults, researchers, clinicians, and designers lack the empirical foundation needed to evaluate the quality of inclusive practices, identify structural barriers to meaningful participation, and develop evidence-informed guidance for responsible co-design. The aim of this scoping review was therefore to identify, map, and critically characterise older adults’ participation in DT processes applied to AI-driven assistive technology development, with explicit attention to the experiential and ethical dimensions of that participation.

## Methods

### Overview

This is a scoping review of the literature reporting on older adults’ participation in, and experiences of, DT processes for AI-driven assistive technology development. Scoping reviews are appropriate when the purpose is to map the extent, range, and nature of a body of evidence and to identify research gaps [21, 22]. Given the emerging, heterogeneous, and cross-disciplinary nature of this evidence base—spanning geriatric medicine, nursing and allied health, gerontology, human–computer interaction (HCI), gerontechnology, and digital health—a scoping review was the most appropriate design [21, 22]. The protocol was prospectively registered on the Open Science Framework (https://doi.org/10.17605/OSF.IO/8W2S5). The review followed Arksey and O’Malley’s [23] five-stage framework with Levac et al. [24] refinements. Reporting adhered to the PRISMA extension for Scoping Reviews (PRISMA-ScR) [25]; the completed PRISMA-ScR checklist is provided as Additional file 1. Consistent with scoping-review methodology, critical appraisal was conducted for descriptive transparency and was not used as a basis for exclusion [21, 23, 26].

### Deviations from the registered protocol

In keeping with the registered commitment to document and justify any departures from the registered plan, the following deviations are reported transparently:

- Screening calibration. The protocol specified a pilot-screening threshold of Cohen’s κ ≥ 0.80, with a repeat pilot on a fresh 10% sample if the threshold was not met. The initial pilot (n = 15; 10% of 146) yielded κ = 0.667 (observed agreement 86.7%), arising from two disagreements at the concept/review (E2/E5) boundary. Rather than repeat the pilot on a new sample, the two disagreements were adjudicated in a structured calibration meeting and both records were excluded by consensus (one technology-acceptance survey with no participatory process, E2; one literature review, E5). This represents a deviation from the registered procedure. We retain the initial κ = 0.667 as the informative reliability estimate; the post-calibration κ = 1.000 is reported only for completeness and is necessarily uninformative once disagreements have been reconciled.
- Search currency and update. The protocol committed to re-running all four databases if more than one month elapsed between the March 2026 search and submission. This condition was triggered. The searches were therefore re-executed across all four databases on 20 June 2026 using the identical strategy; no additional records met all four PCC eligibility blocks, and the final included set remained 15 studies.
- Post-hoc extension of the ethical/inclusion dimension. In the registration, the ethical/inclusion construct corresponds to a single extracted item. For this review it was expanded into a structured set of ethical dimensions (degree of agency; power relations; ageist assumptions; respect for autonomy and dignity; consent processes). This post-hoc extension, permissible in a scoping review, is flagged here as a deviation; the agency-coding scheme is operationally defined below.
- Reporting instrument. The protocol referenced PRISMA-P 2015 (the instrument for systematic-review/meta-analysis protocols) alongside PRISMA-ScR. For this completed scoping review, PRISMA-ScR is the correct and sufficient reporting instrument; PRISMA-P is not used for the review report, and the corresponding PRISMA-P checklist submitted with the protocol has been withdrawn from this manuscript.

### Research questions

The primary research questions, structured using the Population–Concept–Context (PCC) framework [21, 25], were:

- RQ1. What is known from the existing literature about older adults’ participation in DT processes for the development of AI-driven assistive technologies (including the DT phases, roles, and activities involved)?
- RQ2. What experiential and ethical dimensions—including agency, autonomy, dignity, facilitators, barriers, and perceived value—characterise older adults’ participation in those processes?

The PCC framework was operationalised as: **Population**—older adults (aged ≥60 years, per the WHO definition) as primary participants in the DT process; **Concept**—participation in and experiences of DT processes (including operationally equivalent human-centred and participatory design processes), with explicit attention to ethical and experiential dimensions; **Context**—development, evaluation, or improvement of AI-driven assistive technologies intended for use by older adults.

To keep the review tractable and to ensure each question is answered in the Results, the secondary questions were consolidated into three that are reported directly: (S1) which DT frameworks, phases, and activities were applied; (S2) how patterns of participation and inclusion differed across subgroups (technology type; geographic context, high-income vs. LMIC); and (S3) what evidence gaps warrant future primary research. Each research question is mapped to its answering section in Table 4.

### Search strategy

Systematic searches were conducted across four electronic databases—Web of Science, IEEE Xplore, Scopus, and PubMed/MEDLINE—selected to ensure cross-disciplinary capture spanning health sciences and geriatric care (PubMed/MEDLINE), engineering and HCI (IEEE Xplore), and multidisciplinary biomedical and applied research (Scopus, Web of Science) [18]. The search strategy was peer-reviewed by a university librarian using the Peer Review of Electronic Search Strategies (PRESS) guideline [27] before execution. Searches covered January 2015 to the search date and were executed 10–16 March 2026 and re-run on 20 June 2026 (see Deviations). The full PubMed strategy is presented in Table 1; database-specific yields are in Table 2.

**Table 1:** Primary search strategy (PubMed, adapted to all databases)

**Component**	**Search terms**
Population	(“older adult*”[tiab] OR “older people”[tiab] OR “older persons”[tiab] OR “aged”[tiab] OR “ageing population”[tiab] OR “geriatric*”[tiab])
AND Concept	(“design thinking”[tiab] OR “human-centred design”[tiab] OR “human-centered design”[tiab] OR “user-centred design”[tiab] OR “co-design”[tiab] OR “participatory design”[tiab] OR “design sprint*”[tiab] OR “HCDT”[tiab])
AND Technology	(“artificial intelligence”[tiab] OR “AI”[tiab] OR “machine learning”[tiab] OR “conversational agent*”[tiab] OR “chatbot*”[tiab] OR “voice assistant*”[tiab] OR “AI-driven”[tiab] OR “large language model*”[tiab])
AND Context	(“assistive technolog*”[tiab] OR “ageing in place”[tiab] OR “aging in place”[tiab] OR “fall detection”[tiab] OR “cognitive assistance”[tiab] OR “smart home”[tiab] OR “companion robot*”[tiab] OR “social robot*”[tiab])

**Table 2:** Electronic database searches

**Database**	**Search date**	**Search field**	**Records retrieved**
Web of Science	10–16 Mar 2026 (upd. 20 Jun 2026)	Topic (TS=)	53
IEEE Xplore	10–16 Mar 2026 (upd. 20 Jun 2026)	All metadata	3
Scopus	10–16 Mar 2026 (upd. 20 Jun 2026)	TITLE-ABS-KEY	108
PubMed/MEDLINE	10–16 Mar 2026 (upd. 20 Jun 2026)	[tiab]	12
**Total (pre-deduplication)**			**176**

During protocol execution the search strings were refined relative to the registered terms to improve precision and recall at the AI–HCI–gerontechnology intersection: “co-design” and “participatory design” were added to the Concept block, and “companion robot*” and “social robot*” to the Context block, while several lower-yield or off-target registered terms were removed. These changes affect search sensitivity and are reported here for transparency; their effect on yield is addressed in the Limitations.

Search validity was checked against a set of eight pre-specified sentinel (“known-item”) studies expected to be retrievable by the strategy; the strategy retrieved 7 of 8 (threshold ≥6/8 met). The single non-retrieved item was a conference-proceedings record without a structured abstract.

### Eligibility criteria

#### Inclusion

Population: older adults aged ≥60 years (WHO definition) as primary participants; mixed-age samples were eligible if older-adult data were reported separately or if ≥50% of participants were aged ≥60 years. Concept: studies describing or evaluating older adults’ participation in ≥1 DT phase and reporting their experiences, perceptions, roles, or engagement, including ethical and experiential dimensions. Context: development, evaluation, or improvement of AI-driven assistive technologies. Study design: qualitative, quantitative, and mixed-methods empirical studies; protocol papers for primary studies meeting all other criteria. Published in English, January 2015 to search date, full text obtainable.

#### Exclusion

E1: participants not confirmed as older adults (≥60 years) and not reported separately; E2: no DT or operationally equivalent participatory design process; E3: technology not AI-driven assistive technology, or context not technology development; E4: not an empirical primary study (editorial, commentary, theoretical piece); E5: review article; E6: not in English; E7: published before January 2015; E8: full text/abstract not obtainable, or conference-proceedings volume without a retrievable abstract.

### Screening process

**Fig. 1:**
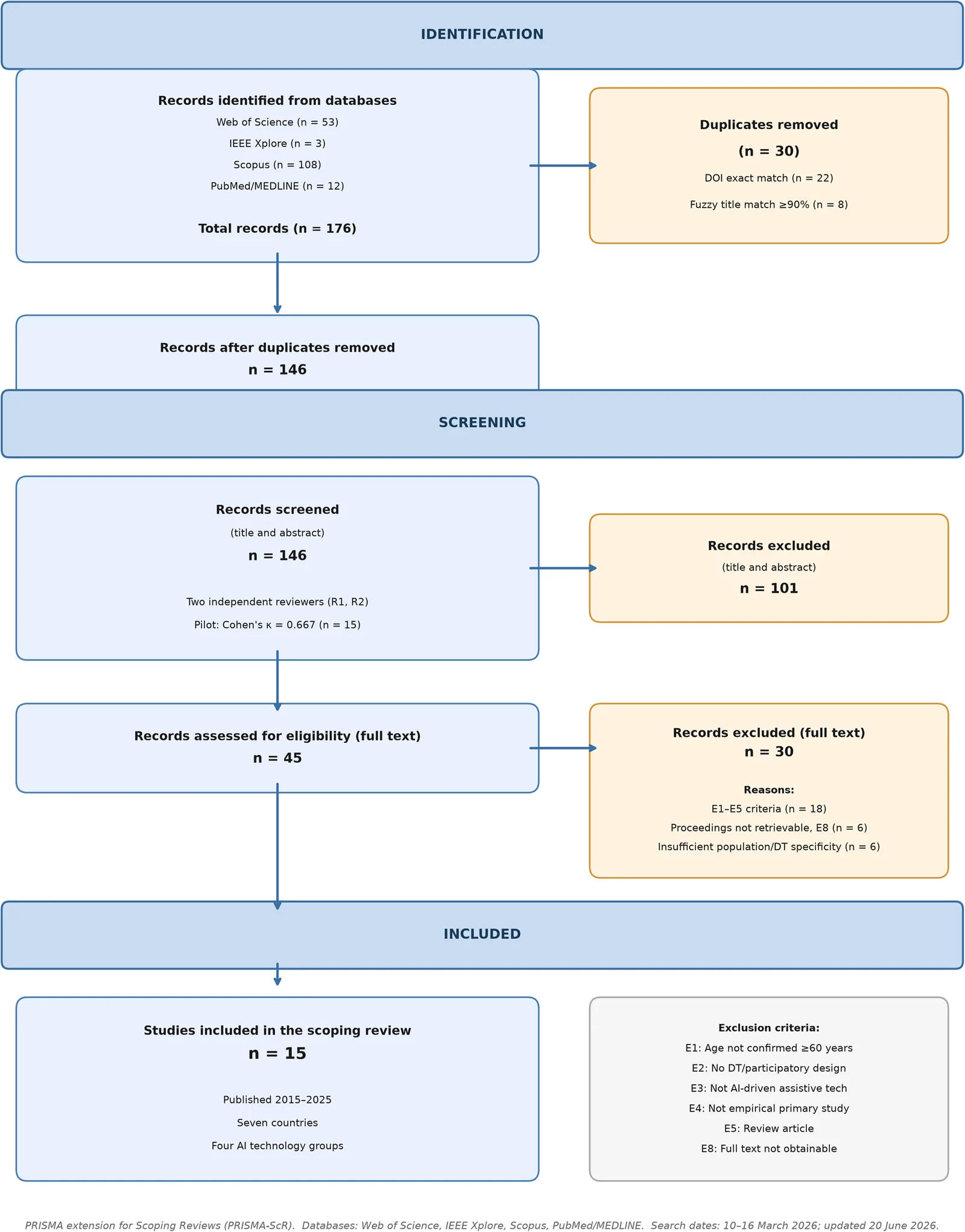
PRISMA-ScR flow diagram of study identification, screening, and inclusion

Records were exported in RIS format, imported into Zotero (v7.x) for deduplication (automated by DOI exact match [n = 22] and manual/fuzzy title match ≥90% via RapidFuzz [n = 8]; 30 removed), and screened in Rayyan (rayyan.ai) with blind mode activated. Deduplication reduced 176 records to 146. Title/abstract and full-text screening were each conducted independently by two reviewers (R1, R2) against all PCC criteria. Pilot screening of a random 10% (n = 15) established inter-rater reliability (Cohen’s κ = 0.667; two E2/E5 boundary disagreements; see Deviations). Discrepancies were resolved by structured consensus; unresolved disagreements were arbitrated by a third senior reviewer. As an additional verification step, all 15 candidate inclusions underwent independent full-text and DOI confirmation by the senior reviewer, which resulted in author-list and DOI corrections to five records. Of 176 records identified, 30 duplicates were removed (n = 146 screened), 101 were excluded at title/abstract, 45 advanced to full text, 30 were excluded at full text (E1–E5, n = 18; proceedings volumes E8, n = 6; manual quality review for insufficient population/DT specificity, n = 6), and 15 studies were included. The PRISMA-ScR flow diagram is presented as Fig. 1.

### Data charting

Data were charted in duplicate by two independent reviewers using a standardised Microsoft Excel form, piloted on five purposively selected studies. Inter-rater agreement for charting met the pre-specified thresholds (≥85% categorical agreement; intraclass correlation coefficient ≥0.75). The charting form (Table 3) was extended with experiential and ethical dimensions that go beyond demographic and study characteristics. To avoid effectiveness-review terminology inappropriate to a scoping review, the form’s categories are described as dimensions/constructs rather than “variables,” and no construct is framed as a “primary outcome”; a scoping review maps rather than estimates outcomes.

**Table 3:** Data-charting form (domains and dimensions)

**Domain**	**Dimensions charted**
Metadata	Author(s); year; title; source; DOI; country; funding; publication type
Study characteristics	Design; theoretical/conceptual framework; DT framework named; sample size; setting; duration
Population	Age range/mean; sex/gender; cognitive and functional status; geographic and cultural context; prior technology experience
AI technology	Type; development stage; primary vs. secondary user role of older adults
DT dimensions	DT phases in which older adults participated; role (co-designer, informant, evaluator); activities; facilitation approach
Experiential dimensions	Lived experience; usability/UX; psycho-affective states (satisfaction, emotional response, ownership); facilitators; barriers
Ethical/inclusion dimensions (extended post-hoc)	Degree of agency (tokenistic/consultative/genuine co-design); power relations; ageist assumptions; respect for autonomy and dignity; consent process; ethics approval
Design outcomes	Tangible design outputs; evidence of older-adult influence on final product; limitations reported

The experiential dimension was defined a priori, drawing on user-experience and lived-experience frameworks, as comprising three distinguishable components: (i) phenomenological/lived experience, grounded in van Manen’s hermeneutic-phenomenological approach to lived experience [28] (how participation was experienced in its own terms); (ii) usability/user experience, grounded in the Hassenzahl and Tractinsky user-experience framework [29] (interaction quality with the technology and the process, including pragmatic and hedonic or emotional qualities); and (iii) psycho-affective states such as satisfaction, emotional response, and sense of ownership, the last grounded in the theory of psychological ownership [30]. The ethical dimension was defined as the conditions and qualities of participation bearing on rights and justice: degree of agency, framed using Arnstein’s ladder of participation [31]; power relations, drawing on Bratteteig and Wagner’s analysis of power and decision-making in participatory design [32]; ageist assumptions, informed by conceptual work on ageism [10] and digital ageism in AI [9]; respect for autonomy and dignity, grounded in the principlist framework of biomedical ethics [33]; and consent processes, informed by process-consent and assent approaches for people with dementia [33, 34]. Where a construct (e.g., agency or ownership) bridges both dimensions, this is treated as a continuum rather than a strict binary. The agency-coding scheme was operationally defined as: tokenistic (older adults consulted symbolically, with no demonstrable influence on design decisions); consultative (older adults provide input that informs, but does not determine, decisions controlled by the design team); and genuine co-design (older adults hold shared decision-making authority over design choices, with documented influence on the final product).

### Critical appraisal

Included studies were appraised in duplicate for descriptive transparency (not for exclusion). Qualitative studies were appraised with the Joanna Briggs Institute (JBI) Critical Appraisal Checklist for qualitative research, and mixed-methods and quantitative studies with the Mixed Methods Appraisal Tool (MMAT) v2018. Disagreements were resolved by discussion. Results are summarised in Table 4.

**Table 4:** Descriptive quality appraisal

**ID**	**Appraisal tool**	**Overall rating**	**Principal limitation noted**
S1	JBI (qualitative)	High	Reflexivity under-reported
S2	JBI (qualitative)	High	Single-site sample
S3	MMAT (mixed)	High	Small validation sub-sample
S4	MMAT (mixed)	High	Limited older-adult role beyond evaluation
S5	JBI (qualitative)	Moderate	Brief methods (conference paper)
S6	MMAT (mixed)	Moderate	Small, single living-lab sample
S7	JBI (qualitative)	High	Influence on final design partly unclear
S8	JBI (qualitative)	High	Empathise-phase only
S9	JBI (qualitative)	Moderate	Brief reporting (conference paper)
S10	JBI (qualitative)	Moderate	Personas-driven; limited direct voice
S11	JBI (qualitative)	Moderate	Heterogeneous multi-site reporting
S12	MMAT (mixed)	Moderate	Needs-analysis emphasis
S13	JBI (qualitative)	Moderate–High	DT tools explicit; sample detail limited
S14	MMAT (mixed)	Moderate	Policy/scenario emphasis over user voice
S15	MMAT (quantitative)	Moderate	Evaluation-only; small N

### Synthesis

Findings were synthesised through inductive narrative thematic synthesis [21, 23, 24]. No meta-analysis was conducted, given heterogeneity of designs and AI technology types. Synthesis proceeded in five steps: (1) descriptive mapping of study characteristics (Table 5); (2) line-by-line inductive coding in NVivo v14, with an iteratively refined codebook and coding memos, and 25% of studies double-coded; (3) clustering of codes into higher-order analytical themes; (4) construction of a thematic synthesis matrix linking themes to contributing studies (Table 6); and (5) evidence-gap mapping (Table 7). The experiential and ethical dimensions informed coding but did not predetermine themes. Each theme was stratified by level of support: well-supported (≥5 studies), moderately supported (2–4 studies), emerging (1 study) [35]. Two pre-specified analyses—by AI technology subtype, and a sensitivity analysis excluding studies that did not explicitly name a DT framework—and a geographic subgroup analysis (high-income vs. LMIC) are reported alongside the main findings.

**Table 5:** Characteristics of included studies (n = 15)

**ID**	**Author (year)**	**Country**	**Design**	**DT/HCD framework**	**AI technology**	**Sample (older adults)**	**DT phases with older-adult participation**	**Role of older adults**	**Agency level**
S1	Irfan et al. (2024) [4]	Sweden	Qualitative co-design	Participatory design/co-design	LLM-based companion robot	28	Empathise; Ideate; Test	Co-designer	Genuine co-design
S2	Irfan et al. (2025) [36]	Sweden	Qualitative, iterative participatory design	Iterative participatory design	LLM (GPT-3.5) conversational robot	34	Empathise; Prototype; Test	Co-designer	Genuine co-design
S3	Mahmood et al. (2025) [37]	USA	Mixed methods (co-design, prototype, in-home)	Five-stage design process	LLM-powered voice assistant	17/10/5	Empathise; Define; Ideate; Prototype; Test	Co-designer	Genuine co-design
S4	Blavette et al. (2025) [38]	France	Mixed-methods longitudinal (3 waves)	Iterative user-centred design	LLM-integrated socially assistive robot	97	Empathise; Test	Evaluator/informant	Consultative
S5	Dawood et al. (2024) [39]	Saudi Arabia	Qualitative co-design (focus groups)	Co-design (3 focus groups)	Voice-based conversational agent	3 focus groups	Empathise; Ideate	Co-designer	Consultative
S6	Wargnier et al. (2015) [40]	France	Prototype evaluation, iterative co-design living lab	Co-design living lab	ECA with attention monitoring	Older adults w/cognitive impairment	Empathise; Prototype; Test	Informant/evaluator	Consultative
S7	Tiersen et al. (2021) [7]	UK	Qualitative iterative UCD (9 substudies)	User-centred/participatory design	Smart-home AI sensing	Dementia households (interviews + workshops)	Empathise; Define; Test	Co-designer/informant	Consultative
S8	Wang et al. (2019) [2]	USA	Qualitative (focus groups)	User-centred design	Smart-home AI technologies (general)	31	Empathise	Informant	Consultative
S9	Bray et al. (2023) [41]	USA	Qualitative co-design (focus group + think-aloud)	Human-centred/participatory design	Social robot for dementia	12	Empathise; Ideate; Test	Co-designer	Consultative
S10	Gaspar et al. (2018) [42]	Brazil/Italy	Design research (participatory design, personas, semiotics)	Participatory design	IoT smart home with AI	Participatory design study	Empathise; Define; Ideate	Informant	Tokenistic→consultative
S11	Loi (2019) [43]	USA/Global	Qualitative design research (multi-site, participatory)	Participatory design	Ambient computing/intelligent systems	Multi-site global study	Empathise; Ideate	Informant	Consultative
S12	Portet et al. (2017) [44]	France	Design research (user-needs + co-design)	User-centred/co-design	AI-enabled smart-home systems	User-needs study	Empathise; Define	Informant	Consultative
S13	Suppipat et al. (2020) [45]	Taiwan	Design research (contextual design)	Contextual design (HCD) with explicit DT tools	Smart-home AI intergenerational service	Contextual design study	Empathise; Define; Ideate; Prototype	Co-designer	Consultative→genuine
S14	Bricout et al. (2022) [46]	USA	Mixed methods (inclusive participatory design)	Inclusive/participatory design + scenario planning	AI-enabled assistive technologies (general)	Inclusive design + policy	Empathise; Define	Informant	Consultative
S15	Garg et al. (2025) [47]	India	Prototype evaluation (UCD)	User-centred design	On-device AI exercise monitoring + wearable	15	Test	Evaluator	Tokenistic

**Table 6:** Thematic synthesis matrix (themes × contributing studies)

**Theme**	**Representative codes**	**Contributing studies**	**Support level**
1. Uneven participation across DT cycle	Empathise-heavy; test-heavy; thin ideate/prototype; informant vs. co-designer	S1–S9, S11–S15	Well-supported (14)
2. Facilitators and barriers	Trust over time; mixed expertise; flexible formats; familiar settings; jargon; one-off sessions; missing accommodations	S2, S4, S5, S6, S7, S8, S9, S11, S14, S15 (+S1, S3)	Well-supported (12)
3. Continuum of agency	Tokenistic; consultative; genuine co-design; documented influence	S1–S15	Well-supported (15)
4. Structural/ethical conditions	Autonomy/dignity; ageism in facilitation; consent/assent; capacity; surrogate involvement	S4, S6, S7, S8, S9, S11, S13, S14	Well-supported (8)

**Table 7:** Evidence-gap map

**Dimension**	**Well-covered**	**Under-represented gap**
DT phase	Empathise; Test	Ideate; Prototype (sustained co-design)
Population	Cognitively intact, urban older adults	Cognitive impairment as active co-designers; frailty; rural residence
Geography	High-income countries	LMICs (esp. low-income); culturally situated co-design
Equity reporting	—	Sex/gender-disaggregated findings; intersectional analysis
Agency	Consultative involvement	Genuine cross-phase co-design authority

## Results

### Selection and characteristics of included studies

Searches yielded 176 records (Web of Science n = 53, IEEE Xplore *n* = 3, Scopus n = 108, PubMed/MEDLINE *n* = 12). After deduplication (30 removed), 146 records underwent title/abstract screening (101 excluded), 45 advanced to full-text review (30 excluded), and 15 studies met all inclusion criteria (Fig. 1).

The 15 included studies were published between 2015 and 2025 and were conducted across seven countries (Table 4). High-income settings predominated—Sweden, the USA, France, the UK, Saudi Arabia, Brazil/Italy, and Taiwan—with one study from India; no study was conducted in a low-income country. AI technologies clustered into four groups: LLM-based conversational agents and companion robots (S1–S5), embodied conversational agents and social robots (S6, S9), smart-home/ambient AI sensing (S7, S8, S10, S11, S12, S13), and AI-enabled wearable/mobility-and-fitness systems (S14, S15). Designs were predominantly qualitative or mixed-methods participatory studies. Sample sizes of older adults, where reported, ranged from 12 to 97.

### Quality appraisal

Descriptive appraisal (Table 5) indicated generally moderate-to-high methodological quality among the peer-reviewed qualitative and mixed-methods studies, with the most consistent limitations being incomplete reporting of reflexivity (researcher positionality) and of the influence of older adults on final design decisions. Conference-proceedings studies tended to report methods more briefly. No study was excluded on quality grounds, consistent with scoping-review methodology.

Mapping of research questions to the sections that answer them: RQ1 (DT phases/roles)—Theme 1 and Table 4; RQ2 (experiential/ethical dimensions)—Themes 2–4, the *Continuum of agency*, and Table 6; S1 (frameworks)—Table 4; S2 (subgroups)—*Subgroup and sensitivity analyses*; S3 (gaps)—*Evidence gaps* and Table 7.

### Thematic synthesis

Inductive coding generated four analytical themes; their relationship to the underlying codes and contributing studies is presented in the thematic synthesis matrix (Table 6).

#### Theme 1—Uneven participation across the DT cycle (well-supported; 14 studies)

Older adults’ participation clustered in the *empathise* phase (immersion, interviews, lived-experience accounts) and the *test* phase (usability evaluation), with comparatively limited involvement in *ideate* and *prototype*. Older adults were more often positioned as informants or evaluators than as co-designers with decision-making authority (S4, S6, S8, S11, S12, S14, S15), although a minority of studies achieved sustained, cross-phase co-design (S1, S2, S3, S13).

#### Theme 2—Facilitators and barriers to meaningful participation (well-supported; 12 studies)

*Facilitators* included clear, accessible communication; longitudinal engagement allowing trust to develop (S2, S4, S7); pairing clinical/gerontological and design expertise within facilitation teams (S4, S6, S7); flexible session formats accommodating sensory, cognitive, and mobility considerations (S5, S6, S9); and embedding sessions in familiar community or care settings (S7, S8). *Barriers* included short, one-off engagements (S15); ageist facilitation assumptions that pre-empted older adults’ contributions (S8, S11, S14); technical jargon; over-reliance on screen-based prototypes; and absence of accommodations for fatigue, vision, hearing, and cognition (S6, S9).

#### Theme 3—A continuum of agency from tokenism to co-design (well-supported; all 15 studies)

Coded against the operational scheme, three studies reflected genuine co-design (S1, S2, S3), with S13 transitioning from consultative toward genuine co-design; the majority were consultative, and two reflected tokenistic involvement (S10 early phases; S15). Agency was higher where older adults participated across multiple phases and held documented influence over design decisions, and lower where involvement was confined to a single phase (typically *test*).

#### Theme 4—Structural and ethical conditions shaping inclusion (well-supported; 8 studies)

Respect for autonomy and dignity, the presence or absence of ageist assumptions, and consent practices varied considerably and were inconsistently reported. Studies engaging older adults with cognitive impairment (S6, S7, S9) raised—but rarely resolved—questions of capacity, surrogate involvement, and assent.

### Subgroup and sensitivity analyses

By AI technology subtype. Genuine co-design was most evident among LLM-based conversational systems (S1, S2, S3), whereas smart-home/ambient sensing studies more often confined older adults to empathise/needs-analysis roles (S8, S11, S12). By geographic context (high-income vs. LMIC). Fourteen of 15 studies were conducted in high-income settings; the single middle-income study (S15, India) was evaluation-only with tokenistic agency, and no low-income-country study was identified—precluding meaningful LMIC comparison and itself constituting a key finding. Sensitivity analysis (excluding studies not explicitly naming a DT framework). Restricting to studies that explicitly named DT/HCD/co-design did not change the four themes or the overall direction of findings; the empathise/test concentration and the predominance of consultative agency persisted.

### Evidence gaps

Evidence-gap mapping (Table 7) identified the most pronounced gaps as: older adults with cognitive impairment as *active* participants (rather than subjects); LMIC and rural contexts; sex/gender-disaggregated reporting; and sustained participation across the *ideate* and *prototype* phases.

### Answers to the research questions

RQ1 (older adults’ participation in DT processes: phases, roles, and activities). Across the 15 included studies, older adults’ participation was real but unevenly distributed across the DT cycle. Participation clustered in the empathise phase (immersion, interviews, and lived-experience accounts) and the test phase (usability evaluation), with comparatively limited involvement in the ideate and prototype phases (Theme 1; Table 6). Older adults were most often positioned as informants or evaluators rather than as co-designers holding decision-making authority; only three studies (S1, S2, S3) achieved sustained, cross-phase co-design, with S13 transitioning toward it (Theme 3; Table 4). The activities that facilitated participation were clear and accessible communication, longitudinal engagement allowing trust to develop, pairing clinical and design expertise within facilitation teams, flexible session formats, and embedding sessions in familiar community or care settings; the main barriers were short one-off engagements, ageist facilitation assumptions, technical jargon, and unmet needs for fatigue, vision, hearing, and cognitive accommodation (Theme 2). By technology subtype, genuine co-design was most evident among LLM-based conversational systems, whereas smart-home and ambient-sensing studies more often confined older adults to empathise or needs-analysis roles.

RQ2 (experiential and ethical dimensions: agency, autonomy, dignity, facilitators, barriers, and perceived value). The experiential dimension showed that participation was experienced most positively when it was sustained over time and when older adults sensed genuine influence over design decisions, fostering satisfaction, emotional engagement, and a sense of ownership; tokenistic or one-off involvement, by contrast, produced frustration and weak perceived value. The ethical dimension revealed a continuum of agency from tokenistic consultation (S10 early phases; S15) through consultative involvement (the majority of studies) to genuine co-design (S1, S2, S3, with S13 transitioning), with agency highest where older adults participated across multiple phases with documented influence on design decisions (Theme 3). Respect for autonomy and dignity, ageist assumptions in facilitation, and consent practices varied considerably and were inconsistently reported; studies engaging older adults with cognitive impairment (S6, S7, S9) raised unresolved questions of capacity, surrogate involvement, and assent (Theme 4). The facilitators and barriers bearing on both dimensions are summarised in Theme 2 and Table 6.

## Discussion

This scoping review mapped how older adults participate in design thinking processes for AI-driven assistive technology and characterised the experiential and ethical dimensions of that participation. It addressed a timely convergence of three forces of direct relevance to geriatric research and practice: the accelerating integration of AI into assistive technology that older adults encounter in everyday care; the global imperative of supporting an ageing population; and the growing consensus—across international policy, geriatric care standards, and digital-health implementation guidance—that meaningful end-user participation is essential to producing technologies that older adults can and will use [1, 3, 11–14].

The contribution of this review is complementary to methodologically focused reviews of participatory design. Whereas prior reviews—including Cho et al. [3]—established *whether* older adults were included in design processes, this review examined *how* that inclusion was experienced and how ethically substantive it was. The differentiation from Cho et al. [3] (no DT lens; no analysis of subjective experience within DT-specific processes; no explicit ethical–experiential framing) is, on its own, sufficient to establish the review’s distinct contribution; we therefore characterise this as an early and explicit experiential–ethical mapping rather than claiming primacy.

Several findings carry implications for geriatric research and practice, each grounded in study-level evidence. First, the concentration of participation in the *empathise* and *test* phases (Theme 1; Table 6) indicates that current practice often falls short of co-design ideals; substantive involvement in *ideate* and *prototype* was the exception (S1, S2, S3, S13) rather than the rule. For technologies intended to support independence and quality of life in later life, deeper involvement across the cycle is needed for tools to reflect older adults’ goals and contexts. Second, ageist facilitation assumptions—older adults positioned as novices or passive informants (Theme 2; S8, S11, S14)—undermine the empathic foundation on which DT depends. Geriatric clinicians, nurses, and care professionals have important roles in co-facilitating sessions, validating older adults’ contributions, and challenging ageist framings in design teams.

Third, the evidence gap concerning older adults with cognitive impairment is particularly consequential and warrants emphasis as the principal gap identified. People living with dementia and mild cognitive impairment are among the most likely beneficiaries of well-designed AI assistive technology, yet they remain among the least represented as *active* participants. Even studies of technologies explicitly intended for this population engaged them chiefly as evaluators or through proxies (S6, S7, S9). Advancing their participation requires concrete inclusive methods: communication aids and simplified, multimodal prototypes; process consent and ongoing assent procedures suited to fluctuating capacity; surrogate and dyadic (care-partner) involvement protocols with appropriate safeguards that preserve the older adult’s voice rather than substituting it; shorter, repeated sessions to manage fatigue; and longitudinal engagement that builds familiarity and trust. These are not peripheral accommodations but prerequisites for ethically valid co-design with this group, and they represent a priority for methodological development and reporting standards.

Fourth, the under-representation of LMIC contexts (Table 7; only S15 outside high-income settings) is both a scientific and an equity concern. Population ageing and AI adoption are accelerating most rapidly in these settings, where contextually appropriate co-design is essential to avoid imposing technologies designed for high-income care ecologies onto markedly different ones. This is directly relevant to the Indonesian context that motivated the review.

We acknowledge a broader structural reality raised in peer review: product design is frequently driven by industry and manufacturing priorities, and shifting toward genuine consumer participation requires advocacy that extends beyond any single review. We do not claim that mapping the literature will, by itself, change industrial practice. Rather, the value of this synthesis is to supply the evidence base that such advocacy requires—documenting where participation is shallow, which populations are excluded, and which facilitation practices distinguish tokenistic from genuine co-design—so that researchers, clinicians, funders, and policymakers can hold development processes to account and so that emerging regulatory instruments (e.g., the EU AI Act) can be operationalised with older adults rather than merely on their behalf.

### Limitations

Restriction to English-language publications may have excluded relevant studies from continental Europe, East Asia, and Latin America [45]. Limitation to four databases may have missed some HCI and engineering conference proceedings; the comparatively low IEEE Xplore yield (n = 3), despite the cross-disciplinary aim, suggests that engineering-venue coverage may be incomplete, and future updates could add the ACM Digital Library and Scopus/IEEE conference indexes. The four-block conjunction restricted largely to title/abstract fields favours precision over sensitivity, which—together with the modest pre-deduplication yield (*n* = 176)—may have under-retrieved some eligible work; the sentinel-study check (7/8 retrieved) provides partial reassurance. Inclusion of operationally equivalent DT processes increased sensitivity but reduced specificity; the pre-specified sensitivity analysis addressed this and did not alter the themes. As a scoping review, this study did not produce pooled effect estimates or formally assess risk of bias across studies; its value lies in mapping the experiential and ethical landscape to prioritise future primary research.

## Conclusions

Meaningful participation of older adults in design thinking for AI-driven assistive technology is important—methodologically and ethically—for producing technologies that older adults can use, want to use, and benefit from. The current evidence base maps a landscape in which participation is real but uneven: concentrated in early and late DT phases, more often consultative than fully co-designed, and shaped by ageist assumptions and structural barriers that limit the depth of older adults’ agency. Older adults with cognitive impairment and those living in low- and middle-income countries remain particularly under-represented. To advance inclusive co-design aligned with healthy-ageing goals, geriatric researchers, clinicians, designers, and policymakers should invest in longitudinal partnerships with older adults, dismantle ageist facilitation defaults, build and report inclusive methods for older adults with cognitive impairment, and prioritise primary research in the contexts where the intersection of ageing and AI is emerging most rapidly.

## Supplementary Information

Supplementary Material 1.

## Data Availability

The datasets supporting the conclusions of this article are available in the Open Science Framework (OSF) repository (https://doi.org/10.17605/OSF.IO/8W2S5). To enable assessment during peer review, the supporting materials—search-result exports (RIS), the Zotero library, Rayyan screening-decision exports (CSV), the data-extraction workbook (XLSX), the inter-rater reliability records, and the quality-appraisal results—have been made available to editors and reviewers via a confidential reviewer-access link on OSF, and will be made fully public upon acceptance. The identical statement has been entered in the submission system.

## Abbreviations

ACM: Association for Computing Machinery
AI: artificial intelligence
CRPD: Convention on the Rights of Persons with Disabilities
DT: design thinking
ECA: embodied conversational agent
EU: European Union
HCI: human–computer interaction
ICC: intraclass correlation coefficient
IEEE: Institute of Electrical and Electronics Engineers
JBI: Joanna Briggs Institute
LLM: large language model
LMIC: low- and middle-income country
LPDP: Indonesian Endowment Fund for Education (Lembaga Pengelola Dana Pendidikan)
MMAT: Mixed Methods Appraisal Tool
OSF: Open Science Framework
PCC: Population–Concept–Context
PRESS: Peer Review of Electronic Search Strategies
PRISMA-ScR: Preferred Reporting Items for Systematic reviews and Meta-Analyses extension for Scoping Reviews
RQ: research question
UCD: user-centred design
UN: United Nations
UNESCO: United Nations Educational, Scientific and Cultural Organization
WHO: World Health Organization
WoS: Web of Science
