# Supplementary material for: Older adults’ participation and experiences in design thinking for AI-driven assistive technology: a scoping review
Source: BMC Geriatr. 2026 Aug 10;26:1393. doi: 10.1186/s12877-026-08096-0 (PMC13746754; doi:10.1186/s12877-026-08096-0)
Supplement: Supplementary file 1 [file 12877_2026_8096_MOESM1_ESM.docx]

**Additional file 1: PRISMA-ScR Checklist**

*Older adults’ participation and experiences in design thinking for AI-driven assistive technology: a scoping review*

Reporting checklist based on: Tricco AC, Lillie E, Zarin W, O’Brien KK, Colquhoun H, Levac D, et al. PRISMA extension for scoping reviews (PRISMA-ScR): checklist and explanation. Ann Intern Med. 2018;169(7):467–473. doi:10.7326/M18-0850.

| **Section** | **Item** | **PRISMA-ScR checklist item** | **Reported (Y/N/NA)** | **Location in manuscript** |
| --- | --- | --- | --- | --- |
| **TITLE** | | | | |
| Title | 1 | Identify the report as a scoping review. | Y | Title page; Title |
| **ABSTRACT** | | | | |
| Structured summary | 2 | Provide a structured summary that includes (as applicable): background, objectives, eligibility criteria, sources of evidence, charting methods, results, and conclusions that relate to the review questions and objectives. | Y | Abstract |
| **INTRODUCTION** | | | | |
| Rationale | 3 | Describe the rationale for the review in the context of what is already known. Explain why the review questions/objectives lend themselves to a scoping review approach. | Y | Background |
| Objectives | 4 | Provide an explicit statement of the questions and objectives being addressed with reference to their key elements (e.g., population or participants, concepts, and context) or other relevant key elements used to conceptualise the review questions and/or objectives. | Y | Methods — Research questions (RQ1–RQ2; PCC) |
| **METHODS** | | | | |
| Protocol and registration | 5 | Indicate whether a review protocol exists; state if and where it can be accessed (e.g., a Web address); and if available, provide registration information, including the registration number. | Y | Abstract; Methods — Overview; OSF https://doi.org/10.17605/OSF.IO/8W2S5 |
| Eligibility criteria | 6 | Specify characteristics of the sources of evidence used as eligibility criteria (e.g., years considered, language, and publication status), and provide a rationale. | Y | Methods — Eligibility criteria |
| Information sources | 7 | Describe all information sources in the search (e.g., databases with date ranges, contact with authors to identify additional sources) and the date the most recent search was executed. | Y | Methods — Search strategy; Table 2 |
| Search | 8 | Present the full electronic search strategy for at least one database, including any limits used, such that it could be repeated. | Y | Table 1 |
| Selection of sources of evidence | 9 | State the process for selecting sources of evidence (i.e., screening and eligibility) included in the scoping review. | Y | Methods — Screening process |
| Data charting process | 10 | Describe the methods of charting data from the included sources of evidence (e.g., calibrated forms or forms that have been tested by the team before their use, and whether data charting was done independently or in duplicate) and any processes for obtaining and confirming data from investigators. | Y | Methods — Data charting |
| Data items | 11 | List and define all variables for which data were sought and any assumptions made about any missing or unclear information. | Y | Table 3 (data-charting form: domains and dimensions) |
| Critical appraisal of individual sources of evidence † | 12 | If done, provide a rationale for conducting a critical appraisal of included sources of evidence; describe the methods used and how this information was used in any potential synthesis. | Y | Methods — Critical appraisal (JBI; MMAT; descriptive, not used for exclusion) |
| Synthesis of results | 13 | Describe the methods of handling and summarising the data that were charted. | Y | Methods — Synthesis |
| **RESULTS** | | | | |
| Selection of sources of evidence | 14 | Give numbers of sources of evidence screened, assessed for eligibility, and included in the scoping review, with reasons for exclusions at each stage, ideally using a flow diagram. | Y | Results — Selection and characteristics; Fig. 1 |
| Characteristics of sources of evidence | 15 | For each source of evidence, present characteristics for which data were charted and provide the citations. | Y | Table 4 (characteristics of included studies) |
| Critical appraisal within sources of evidence † | 16 | If done, present data on critical appraisal of included sources of evidence (see Item 12). | Y | Table 5 (descriptive quality appraisal) |
| Results of individual sources of evidence | 17 | For each included source of evidence, present the relevant data that were charted that relate to the review questions and objectives. | Y | Table 4; thematic synthesis Table 6 (study-level mapping) |
| Synthesis of results | 18 | Summarise and/or present the charting results as they relate to the review questions and objectives. | Y | Results — Thematic synthesis; Table 6 |
| **DISCUSSION** | | | | |
| Summary of evidence | 19 | Summarise the main results (including an overview of concepts, themes, and types of evidence available), link to the review questions and objectives, and consider the relevance to key groups. | Y | Discussion |
| Limitations | 20 | Discuss the limitations of the scoping review process. | Y | Discussion — Limitations |
| Conclusions | 21 | Provide a general interpretation of the results with respect to the review questions and objectives, as well as potential implications and/or next steps. | Y | Conclusions |
| **FUNDING** | | | | |
| Funding | 22 | Describe sources of funding for the included sources of evidence, as well as sources of funding for the scoping review. Describe the role of the funders of the scoping review. | Y | Declarations — Funding |

*† Critical appraisal is an optional component of the scoping review process. In this review, quality appraisal was conducted for descriptive transparency only and was not used as a basis for exclusion, consistent with Arksey & O’Malley (2005) and JBI (2020) scoping-review methodology.*
